# Ralstonia mannitolilytica Bacteremia Stemming From Frequent Outpatient Intravenous Injections at a Local Clinic

**DOI:** 10.7759/cureus.47887

**Published:** 2023-10-28

**Authors:** Yasuhiro Kano, Ken Kikuchi

**Affiliations:** 1 Department of Emergency and General Medicine, Tokyo Metropolitan Tama Medical Center, Fuchu, JPN; 2 Department of Internal Medicine, National Hospital Organization Tochigi Medical Center, Utsunomiya, JPN; 3 Department of Infectious Diseases, Tokyo Women's Medical University, Tokyo, JPN

**Keywords:** ralstonia mannitolilytica, iatrogenic complication, excessive medicine, clinics, bacteremia

## Abstract

An 83-year-old, male patient was admitted with primary bacteremia caused by* Ralstonia mannitolilytica*. Before onset, he had been receiving regular injections at a local clinic for abnormal liver function. The present case is the first in which regular injections apparently served as the transmission route for *R. mannitolilytica* causing bacteremia and demonstrates that this disease can occur in clinics as well as hospitals, raising concerns about the hitherto unnoticed risk of excessive or inappropriate treatments at local clinics.

## Introduction

*Ralstonia mannitolilytica *is an aerobic, glucose non-fermentative, gram-negative rod (GNR), a waterborne, aerobic, gram-negative bacillus commonly found in water and soil which can cause opportunistic, healthcare-associated infections mostly via contaminated solutions (e.g., water for injection and saline) or auxiliary instruments [1-4]. For example, outbreaks of *R. mannitolilytica* bacteremia are typically reported in patients undergoing hemodialysis [5-7]. Common clinical manifestations of *R. mannitolilytica *infection are bacteremia and sepsis, but osteomyelitis [8], endocarditis [9], and meningitis [10] have also been reported. Almost all cases of *R. mannitolilytica* infection occur in the hospital setting and immunocompromised hosts.

Herein, we present a rare case of *R. mannitolilytica *bacteremia that was strongly suspected of arising from frequent injections in a single patient attending a local clinic. This case demonstrates that *R. mannitolilytica* infection outbreaks can occur in clinics as well as hospitals and raises concerns about the hitherto overlooked risk of excessive or inappropriate treatments in local primary care facilities.

## Case presentation

**Figure 1:**
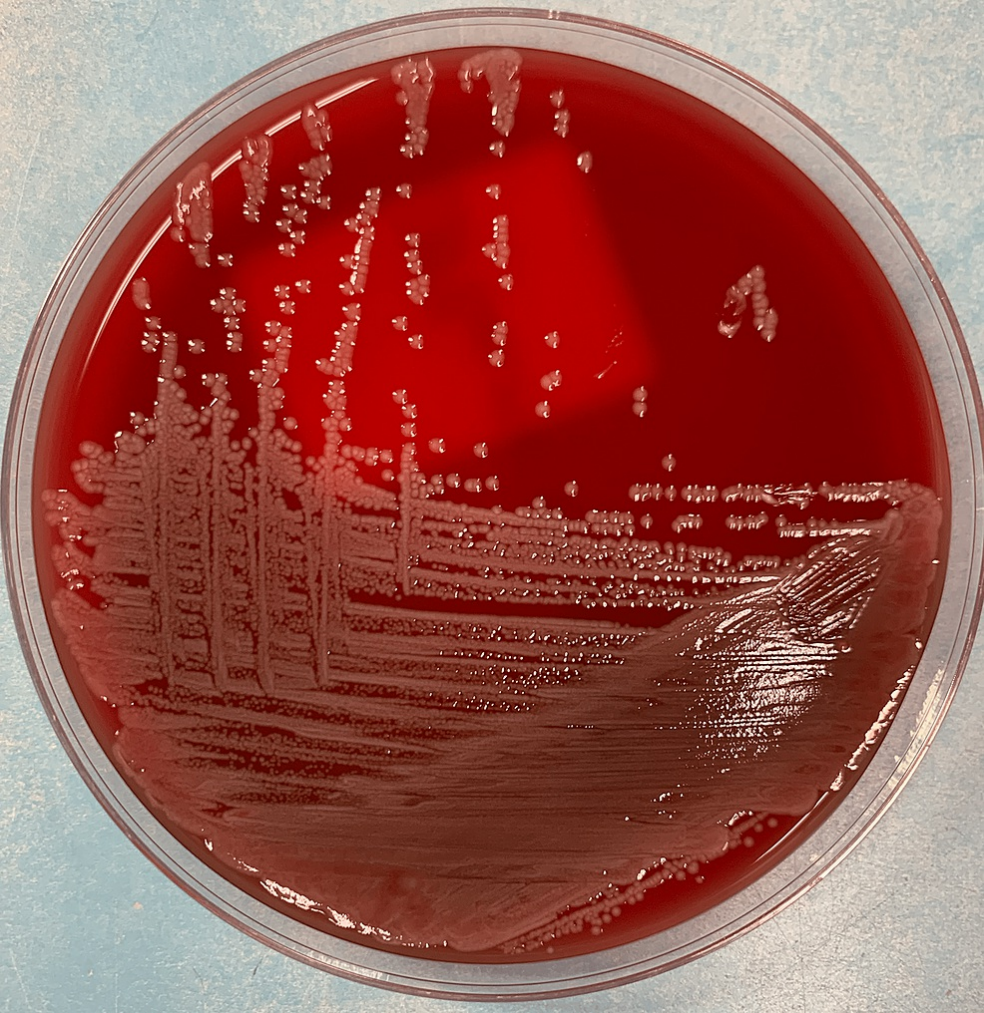
Gross findings of Ralstonia mannitolilytica colonies.

**Figure 2:**
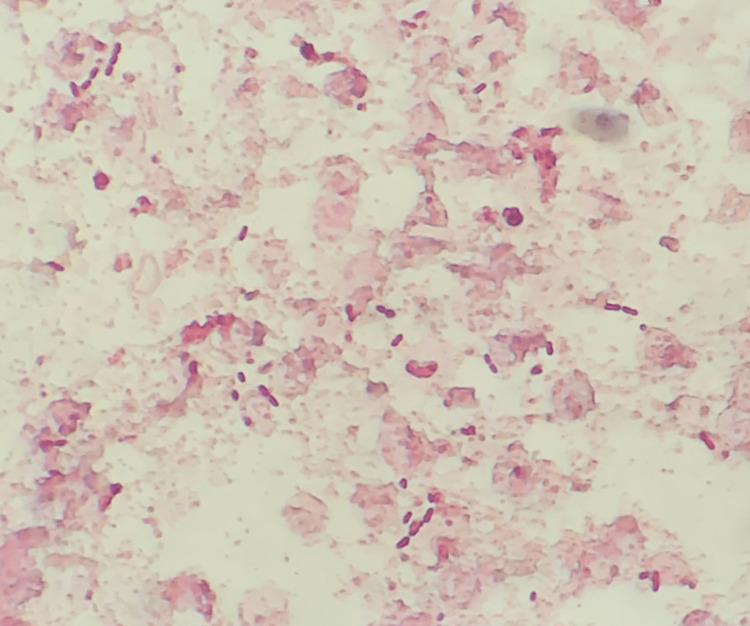
Gram staining of the blood culture showing gram-negative rods.

An 83-year-old, male patient presented with a one-day history of fever and impaired consciousness. His medical history included hypertension, dyslipidemia, hyperuricemia, type 2 diabetes mellitus, complete atrioventricular block with implantation of a permanent pacemaker, and mild abnormal liver function due to nonalcoholic fatty liver disease. His medication included telmisartan, carvedilol, trichlormethiazide, rosuvastatin, ezetimibe, allopurinol, vildagliptin, and metformin. Moreover, he had been receiving regular injections of a monoammonium glycyrrhizinate, glycine, and L-cysteine combination every other day at a local clinic for his abnormal liver function and had received his last injection two days before the current presentation. He had no history of exposure to other medical facilities. His vital signs were temperature 39.7°C (100.9°F), heart rate 102 beats per minute, blood pressure 135/70 mm Hg, and respiration rate 28/min. His level of consciousness was E4V4M6 on the Glasgow Coma Scale. Physical and neurological examination findings were unremarkable. Laboratory tests revealed mildly elevated inflammatory markers, including leukocytes 14.2 (normal 3.3-8.6) × 109/L and C-reactive protein 9.72 (< 0.15) mg/dL. Urinalysis showed no pyuria. A cerebrospinal fluid test, thoracoabdominal computed tomography, and transthoracic echocardiography were normal. His vital signs quickly stabilized with fluid resuscitation only, and he was admitted for watchful waiting without antimicrobial therapy. On hospital Day 2, he defervesced, and his consciousness status normalized. On hospital Day 3, two sets of blood cultures taken on admission returned positive for GNRs (Figures 1, 2).

After performing two more sets of blood cultures, empirical intravenous cefepime 1 g three times daily was begun. Later, these repeat blood cultures returned negative, indicating transient bacteremia. The GNRs were subjected to mass spectrometry (MALDI Biotyper; Bruker Daltonics, Billerica, MA, USA) and 16S ribosomal RNA gene sequencing, which identified *Ralstonia mannitolilytica* (1445/1449 bp identical (99.7%) to the *R. mannitolilytica* strain, LMG 6886T) [4] with a susceptibility profile sensitive to cefepime (Table 1).

**Table 1:** Antimicrobial susceptibility test.

Antimicrobial	Minimum inhibitory concentration	Result
Ampicillin	≥32	Resistant
Piperacillin	64	Intermediate
Cefazolin	≥64	Resistant
Ceftriaxone	2	Susceptible
Cefepime	4	Susceptible
Ceftazidime	>64	Resistant
Cefmetazole	≥64	Resistant
Imipenem	8	Resistant
Meropenem	8	Resistant
Aztreonam	≥64	Resistant
Ampicillin-sulbactam	16	Intermediate
Piperacillin-tazobactam	≥128	Resistant
Tobramycin	≥16	Resistant
Amikacin	≥64	Resistant
Ciprofloxacin	0.5	Susceptible
Levofloxacin	1	Susceptible
Trimethoprim-sulfamethoxazole	≤20	Susceptible

Primary bacteremia caused by *R. *mannitolilytica was finally diagnosed. The patient was discharged after receiving intravenous cefepime for 10 days. He subsequently declined regular injections at the local clinic, and since then, the infection has not recurred.

The primary bacteremia in the present case was thought to be iatrogenic, having been induced by the frequent injections received at the local clinic. Our inquiry about the method of administering injections at the clinic revealed that cotton balls soaked in ethanol and stored in a glass jar were being used to disinfect the skin before injections. While the concentration of the ethanol being used at the clinic is unknown, the tolerance of *R. mannitolilytica* for ethanol was tested by subjecting the organism to various ethanol concentrations in 10% increments. The results demonstrated that the bacterium was able to grow in the presence of ethanol with a concentration as high as 20% [11]. Because no other portal of entry was evident in this case, it was hypothesized that the organism entered the bloodstream via contaminated medical instruments. However, confirmation was not possible because the clinic refused our request for a bacteriological examination of the medical instruments.

## Discussion

Most cases of *R. mannitolilytica* bacteremia reportedly occur as hospital outbreaks [12]. To the best of our knowledge, there are no reports of bacteremia caused by *R. mannitolilytica* stemming from frequent, outpatient, injection therapy in local clinics although an outbreak was reported in oncology outpatients attending a day ward in an Italian hospital where multidose saline bottles used for central venous catheter flushing were identified as the source [13]. Fever and impaired consciousness, the patient’s chief complaints, were considered symptoms of delirium induced by the high-grade fever and sepsis arising from his transient bacteremia.

The route of transmission in the present case was thought to be the injections at the local clinic for the following three reasons: first, there are no reports of community-acquired *R. mannitolilytica* bacteremia unrelated to the healthcare setting; second, the patient had no other medical exposures besides the clinic nor any history of exposure to water or soil that might pose a risk of infection; third, one ampoule of the drug (a monoammonium glycyrrhizinate, glycine, and L-cysteine combination) used at the clinic was analyzed for bacterial contamination but proved to be sterile. While it would have been desirable to investigate the route of contamination, the clinic declined our request for detailed information about injection practices and a bacteriological analysis of the clinic's medical instruments for potential contamination. Nonetheless, the contamination of medical items, such as the cotton wool soaked in ethanol, was thought likely to be the source.

The bacteremia in the present case was transient because the repeated cultures performed before the first antibiotic administration returned negative, suggesting the possibility of similar, undiagnosed events. Monoammonium glycyrrhizinate, glycine, and L-cysteine are sometimes used in Japan when treating liver dysfunction in chronic liver diseases, despite the weak evidence for this practice [14]. The reasons for continuing such an unsupported practice may include disregard for evidence-based medicine, underestimation of treatment complications, and possibly for small, local clinics, the profit motive. To prevent iatrogenic infections like the present case, a monitoring system and protocols need to be established in healthcare institutions, including clinics, and regulations against unnecessary medical treatments need to be strengthened to reduce the risk of treatment-associated complications.

## Conclusions

The present case is the first in which *R. mannitolilytica* bacteremia was apparently caused by frequent injections at a local clinic and demonstrates that bacteremia caused by this pathogen can occur not only in the hospital setting but also in the outpatient clinic, raising concerns about the hitherto overlooked risk of excessive or inappropriate treatments. This case emphasizes the need to improve hygiene management and regulations against unnecessary medical treatments at local clinics.
